# Regulatory coupling between long noncoding RNAs and senescence in irradiated microglia

**DOI:** 10.1186/s12974-020-02001-1

**Published:** 2020-10-28

**Authors:** Anan Xu, Rong Li, Anbang Ren, Haifeng Jian, Zhong Huang, Qingxing Zeng, Baiyao Wang, Jieling Zheng, Xiaoyu Chen, Naiying Zheng, Ronghui Zheng, Yunhong Tian, Mengzhong Liu, Zixu Mao, Aimin Ji, Yawei Yuan

**Affiliations:** 1 Department of Radiation Oncology, Affiliated Cancer Hospital & Institute of Guangzhou Medical University, No 78, Hengzhigang Road, Yuexiu District, Guangzhou, 510095 Guangdong People’s Republic of China; 2 Department of Radiation Oncology, Sun Yat-Sen University Cancer Center, Guangzhou, People’s Republic of China; 3 Department of Pharmacology, Emory University School of Medicine, Atlanta, GA USA

**Keywords:** Microglia, Radiation, Senescence, LncRNA, Inflammation, DNA damage response, Metabolism

## Abstract

**Background:**

Microglia have been implicated in the pathogenesis of radiation-induced brain injury (RIBI), which severely influences the quality of life during long-term survival. Recently, irradiated microglia were speculated to present an aging-like phenotype. Long noncoding RNAs (lncRNAs) have been recognized to regulate a wide spectrum of biological processes, including senescence; however, their potential role in irradiated microglia remains largely uncharacterized.

**Methods:**

We used bioinformatics and experimental methods to identify and analyze the senescence phenotype of irradiated microglia. Western blotting, enzyme-linked immunosorbent assays, immunofluorescence, and quantitative real-time reverse transcription-polymerase chain reaction were performed to clarify the relationship between the radiation-induced differentially expressed lncRNAs (RILs) and the distinctive molecular features of senescence in irradiated microglia.

**Results:**

We found that the senescence of microglia could be induced using ionizing radiation (IR). A mutual regulation mode existed between RILs and three main features of the senescence phenotype in irradiated microglia: inflammation, the DNA damage response (DDR), and metabolism. Specifically, for inflammation, the expression of two selected RILs (ENSMUST00000190863 and ENSMUST00000130679) was dependent on the major inflammatory signaling pathways of nuclear factor kappa B (NF-κB) and mitogen-activated protein kinase (MAPK). The two RILs modulated the activation of NF-κB/MAPK signaling and subsequent inflammatory cytokine secretion. For the DDR, differential severity of DNA damage altered the expression profiles of RILs. The selected RIL, ENSMUST00000130679, promoted the DDR. For metabolism, blockade of sterol regulatory element-binding protein-mediated lipogenesis attenuated the fold-change of several RILs induced by IR.

**Conclusions:**

Our findings revealed that certain RILs interacted with senescence in irradiated microglia. RILs actively participated in the regulation of senescence features, suggesting that RILs could be promising intervention targets to treat RIBI.

**Supplementary Information:**

The online version contains supplementary material available at 10.1186/s12974-020-02001-1.

## Background

Ionizing radiation (IR), a fundamental treatment for intracranial and head-neck cancers, frequently causes irreversible and progressive radiation-induced brain injury (RIBI), especially cognitive deficit, which seriously influences a patient’s quality of life (QoL) during long-term survival [1, 2]. Corticosteroids [3], donepezil [4], and memantine [5] are used to minimize cognitive deterioration; however, their effects are limited. Therefore, a thorough understanding of the pathogenesis of RIBI is urgently required to identify potential targets to prevent RIBI.

Microglia, the effector cells of innate immunity, are responsible for immune defense and brain homeostasis maintenance. In response to certain stimuli, microglia become active and provide beneficial functions that are essential for neuronal survival. However, once their activation passes the threshold of benefit and becomes deleterious, microglia switch to a neurotoxic phenotype and contribute to neuronal damage or death, which ultimately cause neurodegenerative diseases or RIBI [6, 7]. Interestingly, the functional changes of damage-associated molecular patterns (DAMPs)-stimulated microglia in the young adult brain are similar to the changes in normal aged microglia [8]. In addition, the neurotoxic phenotype of microglia exhibits various features of senescence [9, 10].

Cellular senescence, a process that imposes permanent proliferative arrest on cells in response to various stimuli, has emerged as a potentially crucial contributor to aging and age-related diseases. Senescent cells secrete numerous cytokines and matrix metalloproteases (MMPs) and sustain oxidative and genotoxic damage, which consequently contribute to tissue dysfunction and the aging process [11]. Following IR stimulation, the accumulation of DNA damage and the generation of oxidative stress are capable of triggering senescence in normal cells [12–15]. Murine neural stem cells could enter irreversible proliferative arrest, with features of senescence, after in vitro and in vivo IR treatment [12, 13]. In addition, astrocytes acquire senescence characteristics in primary culture conditions and in human brain tissue after exposure to IR [14]. Moreover, IR-induced senescence can be detected in brain microvascular endothelial cells [15]. Recently, it has been reported that the gene expression profile of irradiated primary microglia shares certain similarities with that in normal aging microglia [16]. Therefore, microglia might undergo senescence after IR stimulation; however, the mechanism of this transformation has not been characterized.

Several distinguishing molecular features have been emphasized in senescent cells, including senescence-associated chronic inflammation, a prolonged DNA damage response (DDR), and altered metabolism [11]. Long noncoding RNAs (lncRNAs) comprise a large class of non-protein-coding transcripts with a length >200 bases [17]. Increasing evidence suggests their diverse roles in regulating the three characteristics of senescence. For example, in WI-38 fibroblasts, differentially expressed lncRNAs were compared between young and aged cells. Then, senescence-associated lncRNAs (SAL-RNAs) were identified, several of which are capable of regulating the onset of senescence [18]. In endothelial cells, lncRNA *H19* ameliorates stress-induced senescence by counteracting inflammatory activation [19]. In THP1 macrophages, lncRNA *THRIL* promotes the transcription of the senescence-associated proinflammatory factor, transforming growth factor-alpha (TNFα) [20]. In HeLa cells, suppressor of Ty 6 homolog (SPT6) depletion induced lncRNAs that caused DNA damage, DNA replication stress, and eventually, senescence [21]. In human acute myeloid leukemia cells, lncRNA *ANRIL* inhibits cell senescence by repressing the expression of adiponectin receptor 1 (ADIPOR1), a key regulator of glucose metabolism [22]. Collectively, these data strongly support an essential role for lncRNAs in regulating senescence-associated inflammation, DDR, and metabolism. However, the involvement of lncRNAs in the IR-induced response of microglia remains uncharacterized.

In the present study, we confirmed that IR could induce the senescence of microglia and detected the three main features of irradiated microglia: inflammation, DDR, and metabolism. Altering the expression levels of radiation-induced differentially expressed lncRNAs (RILs) significantly modified the three distinguishing features of senescent microglia, and vice versa. The senescence-dependent regulation of RILs in irradiated microglia provides a method to avoid neuronal toxicity in response to IR.

## Methods

### Cell culture

The mouse microglial cell line BV2 and neuronal cell line HT22 were cultured in Dulbecco’s modified Eagle’s medium (DMEM) mixed with 10% fetal bovine serum (Gibco, Grand Island, NY, USA) and incubated at 37 °C in a 5% CO_2_-humidified incubator.

Primary microglia were derived from mixed glial cultures using the “shaking off” method. Briefly, the brain stem, meninges, and cerebellum from neonatal (P0-1) C57Bl6/J mice were removed, and the remaining parts were chopped and digested using a mixture of papain (30 U/ml, Sigma-Aldrich, St. Louis, MO, USA) and Dnase I (200 μg/ml, Sigma-Aldrich) at 37 °C for 30 min in a 5% CO_2_-humidified incubator. The cell suspension was collected and suspended in culture medium for glial cells (DMEM supplemented with 10% fetal bovine serum) and then cultured in a 75-cm^2^ Falcon tissue culture flask coated with poly-D-lysine (PDL, 10 mg/ml, Sigma-Aldrich) at 37 °C in a 5% CO_2_-humidified incubator. Half of the medium was changed after 8–12 h in culture and every 3 days thereafter, for a total culture time of 10–14 days. Microglia were shaken off from the primary glial cell culture (220 rpm, 37 °C 2 h) with maximum yields between days 10 and 14. The floating cells were collected and seeded into a PDL-pretreated 24-well plate (1 × 10^5^ cells per well in 1 ml) and incubated at 37 °C in a 5% CO_2_-humidified incubator.

### Isolation of adult primary microglia

Adult male C57Bl6/J mice (6–8 weeks old) were used. The mice were sacrificed by anesthesia and perfused with ice-cold Dulbecco’s phosphate-buffered saline (D-PBS). The brain was placed in a culture dish with ice-cold D-PBS. The brain stem, meninges, and cerebellum were removed, and the remaining parts (maximum 500 mg) were digested using 1980 μl of enzyme mix (Miltenyi Biotec, Bergisch Gladbach, Germany). According to the manufacturer’s protocol, the 1980 μl of enzyme mix contained 1950 μl of enzyme mix 1 (enzyme P 50 μl + buffer Z 1900 μl) and 30 μl of enzyme mix 2 (enzyme A 10 μl + buffer Y 20 μl). After removing the debris and red blood cells following the manufacturer’s instructions, the cells were incubated with anti-CD11b (also known as integrin subunit alpha M (ITGAM)) microbeads (Miltenyi Biotec) at 4 °C for 15 min. Magnetic separation was then used to separate the CD11b-positive population from the CD11b-negative population.

### Ionizing radiation

Adherent primary microglia or BV2 cells were irradiated at a distance of 100 cm from the source to the cells using a 6-MV linear accelerator (linac) (Clinac iX, Varian, Palo Alto, CA, USA) or a 225-KV X-ray irradiator (X-RAD 225, Precision X-Ray, Inc., North Branford, CT, USA), then returned to the 37 °C, 5% CO_2_ incubator. The dose-rate was 3 Gy/min for the linac and 2 Gy/min for the X-ray irradiator.

Adult male C57Bl6/J mice (6–8 weeks old) were selected for irradiation. After anesthesia, the head of each mouse was placed in a 2 × 2 cm^2^ treatment field, and a single dose of 10/20 Gy at a dose rate of 3 Gy/min was delivered at a distance of 100 cm from the source to the skin using the Clinac iX. The mice were sacrificed at the indicated time after irradiation.

### Chemicals

Oleic acid (OA) (Sigma-Aldrich) was mixed with 3 ml of 0.1 mM NaOH (Shanghai Macklin Biochemical Co., Ltd, Shanghai, China) and then saponified at 75 °C for 30 min. Next, the OA solution was mixed with 20% bovine serum albumin (BSA) (MRC, Shanghai, China), at 55 °C for 30 min. The stock solution of OA at 10 mM in 10% BSA was then used to make the working solutions.

Lipopolysaccharide (LPS) was purchased from Sigma-Aldrich. Bay-11-7082, SP600125, U0126, and SB203580 were purchased from MedChemExpress LLC (Monmouth Junction, NJ, USA).

### Co-culture of BV2 and HT22 cells

HT22 cells (5.0 × 10^5^ cells per well) were co-cultured with BV2 cells (4 × 10^5^ cells per well) indirectly in 6-well plate chambers (pore size 0.4 μm; Corning, NY, USA) to study the effect of IR-activated microglia on the survival of neuronal cells. In this co-culture system, irradiated BV2 communicated with HT22 through the semipermeable membrane, avoiding direct contact.

### Senescence assays

A senescent cell staining kit (Solarbio, Beijing, China) was used to perform the senescence-associated-β-galactosidase (SA-β-gal) assay according to the manufacturer’s protocol. The senescent BV2 cells (5.0 × 10^5^ cells per well in 6-well plate) or primary microglia (1 × 10^5^ cells per well in a 24-well plate) were identified as blue-staining cells under the microscope (Leica, Wetzlar, Germany). Total cells were counted in five random fields per culture dish to determine the percentage of SA-β-gal-positive cells.

The β-galactosidase activity of primary microglia was measured using a β-galactosidase activity assay kit (Solarbio) following the manufacturer’s protocol. O-nitrophenol was used as the standard solution. The standard, control, and sample were added to a 96-well plate and incubated at 37 °C for 30 min, followed by immunofluorescence. The absorbance of the solutions in the wells was measured at 400 nm. The activity of β-galactosidase was calculated following the manufacturer’s instructions.

### Immunofluorescence

To label p16^INK4A^ (also known as cyclin-dependent kinase inhibitor 2A (CDNK2A)), non-irradiated/irradiated BV2 cells (1 × 10^5^ cells per well in a 24-well plate) on glass coverslips were fixed using 4% paraformaldehyde (PFA) for 15 min and then permeabilized using 0.1% Triton X-100. Next, cells in the coverslips were washed with PBS and blocked in 3% BSA for 30 min. Rabbit anti-p16^INK4A^ primary antibody (Abcam, Cambridge, MA, USA) was used at 1:100 dilution, and goat anti-rabbit AlexaFluor-594 secondary antibodies (Abcam) were used at 1:200 dilution. 2-(4-amidinophenyl)-1H-indole-6-carboxamidine (DAPI); Sigma) staining was used before analysis under a fluorescence microscope (Leica).

To examine the co-distribution of p16^INK4A^ and Iba-1 (ionized calcium-binding adapter molecule 1) in the same sample of primary microglia, a double immunofluorescence procedure was used. After fixation and permeabilization, the adherent non-irradiated/irradiated primary microglia (1.0 × 10^5^ cells per well in a 24-well plate) were blocked in 3% BSA for 30 min and then washed three times with PBS. The primary microglia were then incubated with goat anti-Iba-1 primary antibody (1:100, Novus biologicals, LLC, USA), and rabbit anti-p16^INK4A^ primary antibody (1:100, Abcam) at 4 °C overnight. The primary antibodies were detected by incubation of donkey anti-goat cy3-conjugation secondary antibody (1:200, Proteintech, Rosemont, IL, USA) and goat anti-rabbit AlexaFluor-594 secondary antibody (1:200, Abcam) at room temperature for 1 h. DAPI staining was used before analysis under a fluorescence microscope (Leica).

To examine the co-distribution of p16^INK4A^ and Iba-1 in the same mouse brain slice, a double immunofluorescence procedure was used. Before sacrifice, adult male C57Bl6/J mice were anesthetized and then perfused intracardially with 4% PFA. Brains were postfixed in 4% PFA at 4 °C overnight. Paraffin-embedded brain slides were then baked at 60 °C for 4 h. The primary antibodies included in this study were Goat anti-Iba-1 antibodies (1:100, Novus biologicals) and rabbit anti-p16^INK4A^ antibodies (1:100, Abcam). DAPI staining was used before analysis under a confocal laser-scanning microscope (Olympus, Tokyo, Japan).

To stain lipid droplets, the adherent non-irradiated/irradiated BV2 cells (1.0 × 10^5^ cells per well in a 24-well plate) were washed twice with PBS and then lipiblue (450–480 nm) working solution (Dojindo Molecular Technologies Inc.) was added and incubated at 37 °C for 30 min for immunofluorescence. Total cells were counted in five random fields per culture dish to determine the percentage of lipiblue-positive cells.

The fluorometric terminal deoxynucleotidyl transferase nick-end-labeling (TUNEL) assay was performed following the manufacturer’s instructions (Promega, Madison, WI, USA). Cells were washed twice with PBS after fixing with 4% PFA for 30 min at 4 °C and then permeabilized in 0.2% Triton X-100 for 5 min, followed by rinsing with PBS. After the cells had equilibrated, the nucleotide mix and rTdT buffer were added incubated for 60 min at 37 °C inside a humidified chamber avoiding direct light. Finally, after terminating the reaction using 2× SSC and PBS washing, DAPI staining was used before analysis under a fluorescence microscope (Leica).

### Flow cytometry

The extent of apoptosis was determined by flow cytometry using an Annexin V-APC (Annexin V-Allophycocyanin)/7AAD (7-Aminoactionomycin) assay (Multi sciences biotech, Co., Ltd, Hangzhou, China). According to the manufacturer’s instructions, after co-culture with non-irradiated/irradiated BV2 cells, HT22 cells were washed twice with cold PBS, resuspended, and incubated with Annexin V-APC (dilution 1:40) and 7AAD (dilution 1:40) in the dark for 15 min at room temperature. For each experiment, 1 × 10^5^ cells were analyzed.

### In situ hybridization

Cells on glass coverslips (1.0 × 10^5^ cells per well in a 24-well plate) were fixed using 4% PFA for 30 min and then permeabilized using 0.1% Triton X-100. The coverslips were then washed with PBS and soaked in a pre-hybridization buffer. The in situ hybridization (ISH) digoxigenin-labeled ENSMUST00000190863 or ENSMUST00000130679 probes (Boster, Pleasanton, CA, USA) were reconstituted in hybridization buffer (50% formamide, 10% dextran sulfate, 2× TSSC, 0.01% sheared salmon sperm DNA, and 0.02% SDS), and incubated at 37 °C overnight. Cells were washed twice with 2× saline sodium citrate (SSC) for 5 min at 37 °C, twice with 0.5× SSC for 15 min at 37 °C, and twice with 0.2× SSC for 5 min at 37 °C. After blocking with 5% normal goat serum for 1 h at 37 °C, the cells were incubated with anti-digoxigenin alkaline phosphatase conjugate (Roche Applied Science, Basel, Switzerland) and stained with 5-bromo-4-chloro-3-indolyl phosphate/nitro-blue tetrazolium chloride buffer for 4 h. After DAPI staining, images were collected using a confocal microscope (Olympus).

### RNA-sequencing analysis

Briefly, total RNA was extracted from non-irradiated/irradiated BV2 cells and purified using an RNeasy Mini Kit (Qiagen, Valencia, CA, USA). Next, the RNA samples were reverse transcribed into double-stranded DNA (cDNA) and labeled. RNA-seq analysis was performed by Sagene, Co. (Guangzhou, China) using the Illumina HiSeq™ 2500 platform. After analyzing the significance and false discovery rate (FDR), differentially expressed genes were chosen according to the *P* value threshold and fold change. Functional profiling was performed using Gene Ontology (GO) and Kyoto Encyclopedia of Genes and Genomes (KEGG) databases.

### siRNA transfection

Small interfering RNA (siRNA) targeting ENSMUST00000190863 or ENSMUST00000130679 and scrambled sequence (negative control, NC) were purchased from Generay (Shanghai, China). Cells were seeded on a 6-well plate and grown until they reached 50–60% confluence on the second day. Transfection was performed using Lipofectamine 3000 (Invitrogen Co., Carlsbad, California, US) according to the manufacture’s instruction. The three target sequences for ENSMUST00000190863 were 5′-GAAGCACATATCCACATTA-3′, 5′-GCTAAGGACTAGGCCATAT-3′ and 5′-CCAGAGAACCAAAGAGAAA-3′, respectively. The three target sequences for ENSMUST00000130679 were 5′-GCTTCACGCTTCACGCATA-3′, 5′-CTTCCTTAGTCTACCATCA-3′, and 5′-CAACTTCACGTTTCTCTAA-3′, respectively.

### Construction of a recombinant virus

H1-GFP-puro lentiviral vectors (synthesized by GenePharma) were used to knockdown the expression of ENSMUST00000190863 or ENSMUST00000130679 using short hairpin RNAs (shRNAs). A non-silencing shRNA cloned into the lentiviral vector was used as the negative control. The target sequences for ENSMUST00000190863 and ENSMUST00000130679 were 5′-CCAGAGAACCAAAGAGAAA-3′ and 5′-GCTTCACGCTTCACGCATA-3′, respectively.

### Quantitative real-time reverse transcription PCR (qRT-PCR)

Total RNA was extracted using a Trizol reagent kit (Takara, Dalian, China), according to the manufacturer’s instruction. After reversing transcription using a PrimeScript™ RT reagent kit (Takara), quantitative real-time PCR (qPCR)was executed using a SYBR Premix Ex Taq™ kit (Takara) using gene-specific primers. The 2^-ΔΔCT^ method was used to determine the fold changes. The sequences of the primers are shown in Supplementary Table 1.

### Cell fractionation

Nuclear and cytoplasmic RNA from BV2 cells were fractionated using a purification kit (Norgenbiotek, ON, Canada). According to the manufacturer’s protocol, after washing the cell monolayer with PBS, 200 μl of ice-cold lysis buffer J was added to the 6-well culture plate for 5 min, and then the lysate was transferred to an RNase-free microcentrifuge tube and centrifuged for 10 min at 15000 rpm. Next, 200 μl of buffer SK was added to the supernatant (cytoplasmic RNA fraction) and 400 μl of buffer SK was added to the pellet (nuclear RNA fraction). After vortexing, 200 μl of 100% ethanol was added to each mixture. The mixtures were applied to a spin column, centrifuged for 1 min at 6000 rpm. The flowthrough was discarded, and 400 μl of wash solution A was added to the column, which was centrifuged for 1 min three times. Next, 50 μl of elution buffer E was added to the column, which was centrifuged for 2 min at 2000 rpm and 1 min at 14,000 rpm. The purified RNA sample was subjected to qRT-PCR analysis.

### Western blotting

Cell lysates were harvested at the indicated times using radio-immunoprecipitation assay (RIPA) buffer (Cell signaling technology, CST, Danvers, MA, USA) containing phosphatase inhibitors and proteinase cocktails (Sigma-Aldrich). Protein concentrations were measured using a bicinchoninic acid (BCA) protein assay kit (ComWin Biotech, Beijing, China). Equal amounts of proteins were separated using SDS-polyacrylamide gel electrophoresis, transferred to a polyvinylidene fluoride membrane, followed by reaction with primary antibodies. After reaction with labeled secondary antibodies, the target proteins were visualized using an ECL detection kit (Millipore, Billerica, MA, USA). The primary antibodies included in this study were rabbit anti-gamma H2A histone family member X (γH2A.X) (1:2000, Abcam), rabbit anti-nuclear factor-kappa B p65 subunit (NFκB-P65^S536^) (1:1000, SAB, College Park, MD, USA), rabbit anti-mitogen-activated protein kinase 14 (P38T-180 Y-182 MAPK) (1:1000, SAB), mouse anti-JUN *N*-terminal kinase (JNKT-183 Y-185) (1:1000, CST), rabbit anti-NFκB-P65 (1:1000, CST), mouse anti-P38 MAPK (1:1000, Proteintech, Rosemont, IL, USA), mouse anti-JNK (1:1000, CST), rabbit anti-TP53 (1:1000, p53) (CST), rabbit anti-glyceraldehyde-3-phosphate dehydrogenase (GAPDH) (1:1000, Proteintech), and rabbit anti-β-actin (1:1000, Proteintech).

### Enzyme-linked immunosorbent assay (ELISA)

Cells were seeded into a 6-well plate. Culture supernatants were collected 24 h after IR exposure to detect the concentrations of TNFα, IL-1β, and IL-6. According to the manufacturer’s instructions (Origene, Rockville, MD, USA), 100 μl of standard, control, or samples were added to a 96-well plate, and then 50 μl Biotin-labeled primary antibody was added to each well. After 2 h of incubation and four washes, 100 μl of horseradish peroxidase-conjugated antibody was added to each well and incubated for 30 min in the dark. After washing four times, 100 μl of 3,3′,5,5′-Tetramethylbenzidine (TMB) substrate was added to each well for 30 min. Finally, 100 μl of stop solution was added and the absorbance at 450 nm was determined using a microplate reader.

### Figure preparation and statistics

Figures were prepared using GraphPad Prism 6.0 (GraphPad Software, Inc., La Jolla, CA, USA). Images were edited using Adobe Photoshop CS3 (Microsoft Corp., Redmond, WA, USA). The size of the sample for each sub-group was 5–8 (experiments in cell lines and primary cells were 5–8 wells per sub-group, in vivo experiments using mouse brains were 6 per sub-group), each experiment was repeated at least in triplicate, and the mean ± standard deviation is presented. The Z-score was used for data normalization. Student’s *t* test and one-way analysis of variance (ANOVA) with Bonferroni’s correction were used to compare the statistically significant differences between two groups and multiple groups, respectively. *P* < 0.05 was considered statistically significant.

## Results

### Irradiation induces microglial senescence in vitro

**Fig. 1:**
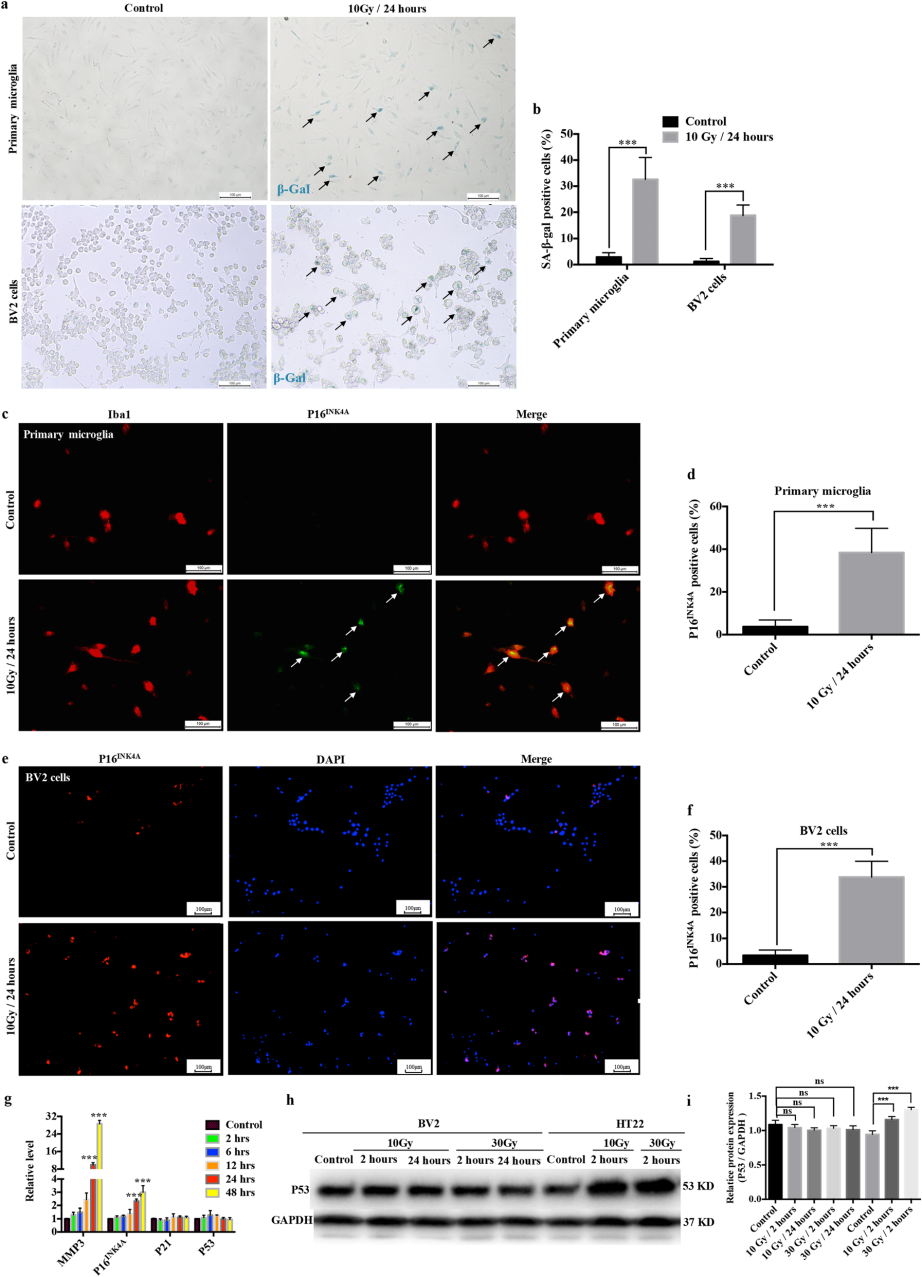
Irradiation induces microglial senescence in vitro. **a** Cytochemical staining for SA-β-gal in primary microglia and BV2 cells at 24 h after 10 Gy linac irradiation. Representative images are shown (scale bar, 100 μm). **b** The ratio of SA-β-gal positive cells is shown as the mean ± SEM; ***P < 0.001, versus the control group. **c** Primary microglia were exposed to a single dose of IR (10 Gy) by linac. Cells were immunostained with anti-Iba-1 (red) and anti-P16^INK4A^ (green) antibodies 24 h after IR. Representative images are shown (scale bar, 100 μm). **d** The ratio of P16^INK4A^ positive primary microglia is shown as the mean ± SEM; ***P < 0.001, versus the control group. **e** BV2 cells were exposed to a single dose of IR (10 Gy) by linac. Cells were immunostained with anti-P16^INK4A^ antibody (red) 24 h after IR. Representative images are shown (scale bar, 100 μm). **f** The ratio of P16^INK4A^ positive BV2 cells is shown as the mean ± SEM; ***P < 0.001, versus the control group. **g** Expression levels of *Mmp3*, *Cdkn2a* (P16^INK4A^), *Cdkn1a* (P21), and *p53* in BV2 cells at different time points after 10 Gy IR by linac were examined using qRT-PCR (mean ± SEM, ***P < 0.001). **h** The protein levels of p53 from BV2 cells and HT22 cells after exposure to different doses at different time points, as examined using western blotting. **i** Histogram of the quantification of the immunoreactive protein bands on the western blots (mean ± SEM, ns, not significant, ***P < 0.001)

It has been demonstrated that IR induces senescence in various cell types in the CNS [12–15]. In addition, certain similarities exist in the gene expression profiles of irradiated primary microglia and normal aging microglia [16]. To characterize cellular senescence in irradiated microglia, we examined the key senescent marker, senescence-associated-β-galactosidase (SA-β-Gal), in primary microglia and BV2 cells at 24 h after a single dose of 10 Gy delivered using a medical linac. The results showed much higher numbers of SA-β-Gal-stained cells after IR stimulation (Fig. 1a, b). Besides SA-β-Gal, the other key tool to identify cellular senescence is the expression of p16^INK4A^, which is a cyclin-dependent kinase inhibitor (CDKi) that serves as a master regulator of cell cycle arrest [23]. Our study showed that the numbers of p16^INK4A^-positive primary microglia and BV2 cells increased dramatically at 24 h after 10 Gy delivered using the linac (Fig. 1c–f).

As presented in Fig. 1g, the level of p16^INK4A^ in BV2 cells began to increase at 24 h after exposure to 10 Gy and was constantly elevated at 48 h. Two parallel yet interacting signaling pathways, p53–p21–retinoblastoma protein (RB) and p16^INK4A^–RB, participate in initiating and maintaining senescence [23]. Strikingly, the expression of p21 and p53 did not increase in the irradiated microglia (Fig. 1g–i). Therefore, we speculated that the p16^INK4A^–RB pathway might be the main functional pathway that mediates senescence in irradiated microglia. In addition, the expression of matrix metalloproteinase 3 (MMP3), a classical marker of senescence [24], also increased dramatically at 24 h after IR (Fig. 1g). These results provided evidence that IR exposure induced microglial senescence in vitro.

### Irradiation induces microglial senescence in vivo

**Fig. 2:**
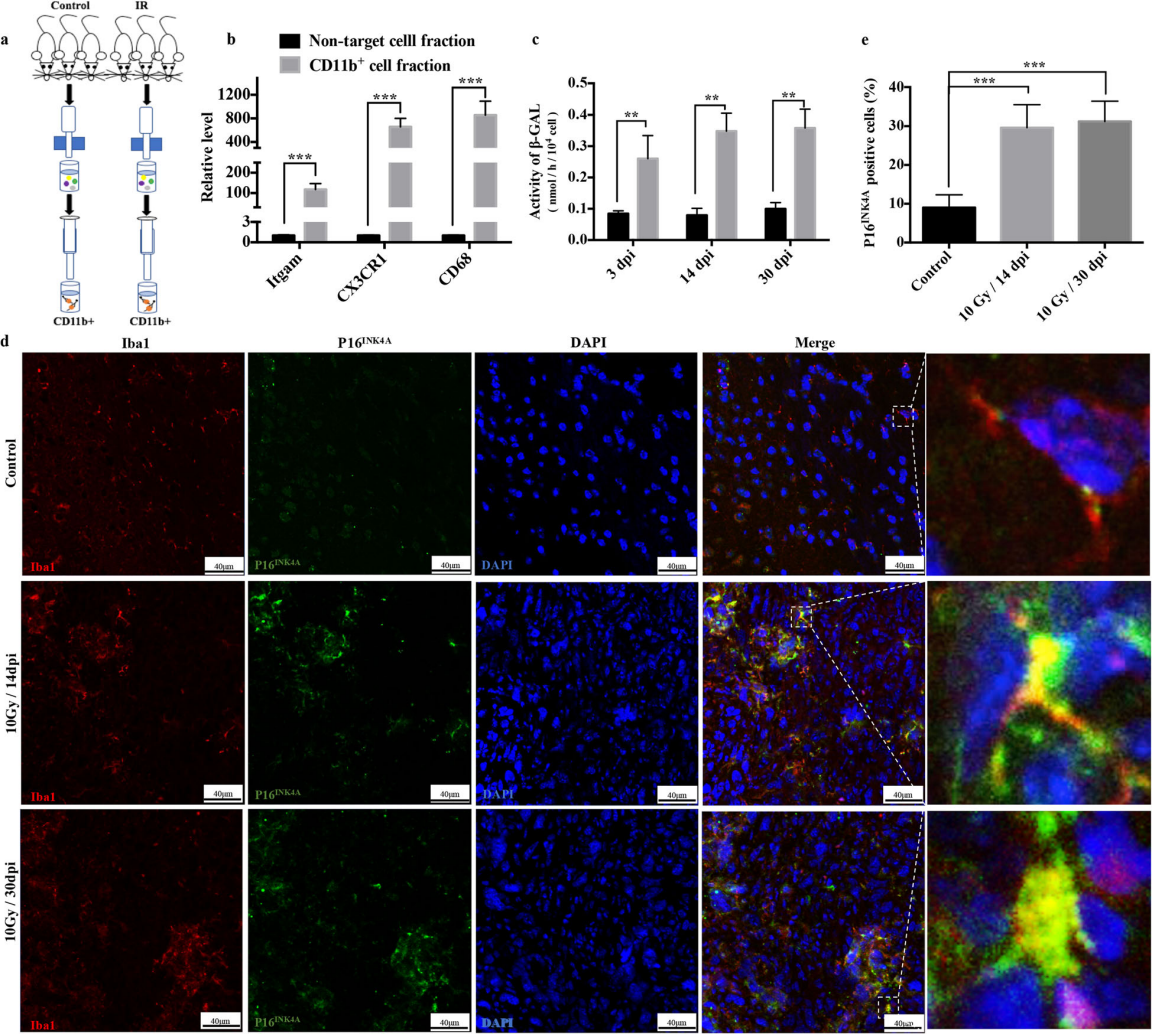
Irradiation induces microglial senescence in vivo. **a** Schematic diagram of the isolation of primary microglia from 6-week-old C57BL/6 J male mice after IR at 20 Gy using a linac. **b** The expression level of microglial markers, including ITGAM, CX3CR1, and CD68, in primary microglia (mean ± SEM, ***P < 0.001). **c** The activity of β-galactosidase of irradiated microglia isolated from C57BL/6 J male mice at different day post-irradiation (dpi) is shown as the mean ± SEM; **P < 0.01, versus the control group. **d** Adult male C57Bl6/J mice were exposed to a single dose of IR (10 Gy) by linac. Representative sections from cortex immunostained with anti-Iba-1 (red) and anti-P16^INK4A^ (green) antibodies at different dpi are shown (scale bar, 40 μm). **e** The ratio of P16^INK4A^ positive microglia in the cortex is shown as the mean ± SEM; ***P < 0.001, versus the control group

To confirm the persistence of microglial senescence after irradiation in vivo, microglia from the cortex and hippocampus of irradiated adult male C57Bl6/J mice were isolated using a magnetic-activated cell sorting (MACS) technique (Fig. 2a). The sorting efficiency is presented in Fig. 2b. The result showed the continuous existence of activated β-galactosidase in isolated microglia at 14 and 30 days post-irradiation (Fig. 2c). The expression of the other key marker, p16^INK4A^, was also upregulated in microglia at 14 and 30 days post-irradiation in mouse brain slices (Fig. 2d, e). These results confirmed that IR induced persistence of senescence markers in microglia in vivo. Collectively, our results demonstrated that microglial senescence could be triggered by IR both in vitro and in vivo.

### The molecular features of senescence in irradiated microglia

**Fig. 3:**
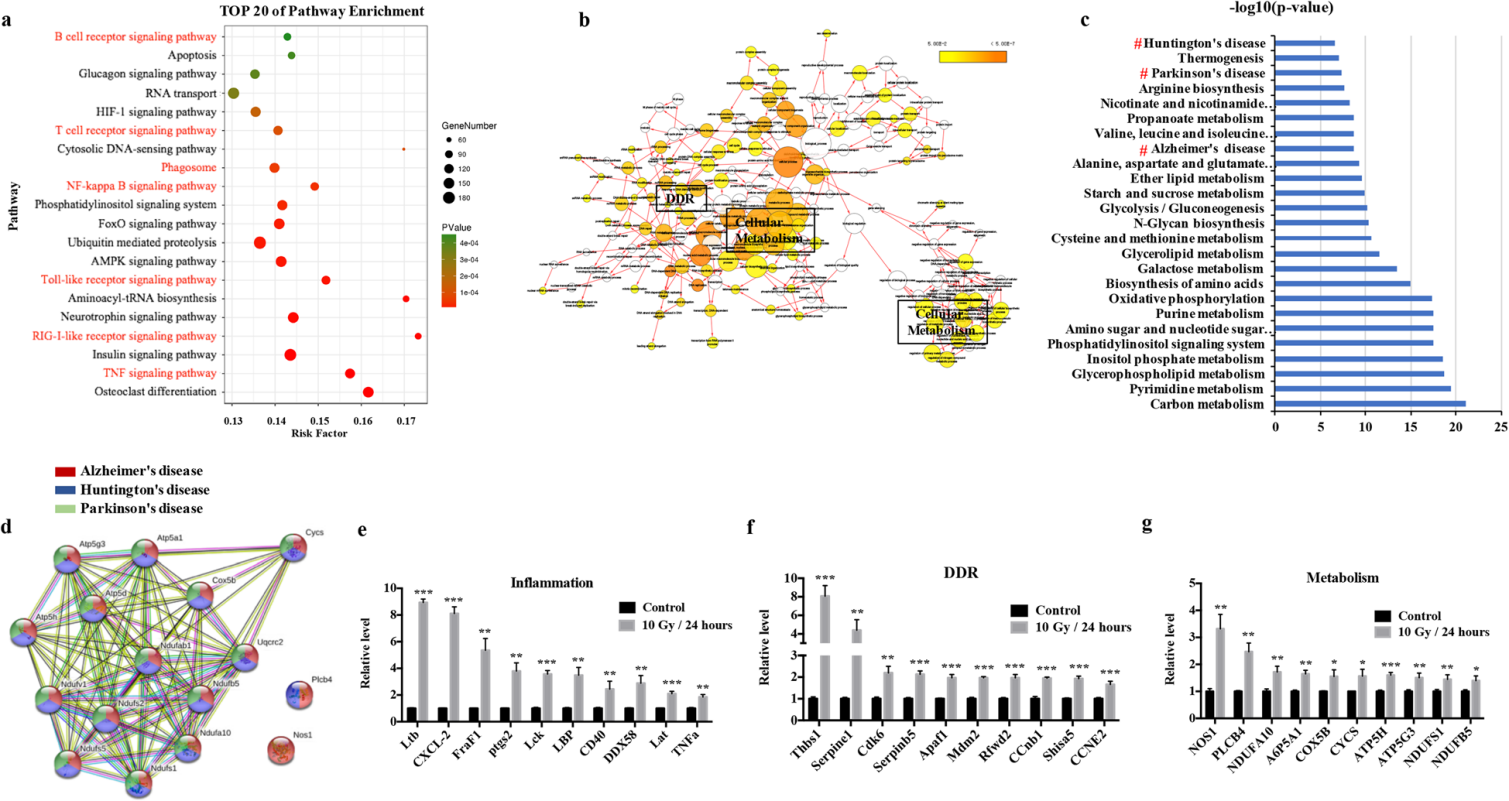
The molecular features of senescence in irradiated microglia. **a** To identify the mRNAs whose expression is altered in irradiated microglia, an RNA-sequencing analysis (RNA-seq) in BV2 cells was performed. BV2 cells were collected 24 h after exposure to 10 Gy using a linac. Pathway analysis (KEGG) of the differentially expressed genes (FDR *q* value <0.05) identified the first 20 pathways that exhibited significant differences. The pathways in red represent those related to inflammation. **b** Biological network analysis using CentiScaPe of IR-induced differentially expressed genes (FDR *q* value <0.05). The size of the nodes is proportional to the gene number, and a darker colored node represents more significant enrichment. **c** KEGG analysis of 316 significantly changed metabolism-related genes (FDR *q* value <0.05) identified the top 25 pathways that exhibited significant differences. Hashes (#) represent neurodegenerative diseases. **d** Network of gene sets related to neurodegeneration in the irradiated microglia gene signature. **e-g** Expression levels of genes related to inflammation, the DDR, and metabolism in BV2 cells, as examined using qRT-PCR at 24 h after 10 Gy by linac (mean ± SEM, *P < 0.05, **P < 0.01, ***P < 0.001)

To identify the molecular features in irradiated microglia, we performed RNA-seq analysis based on non-irradiated/irradiated BV2 cells. Microglia are dysfunctionally activated by IR, which triggers inflammation and stress [25]. As expected, pathway analysis and functional annotation showed that seven out of the top 20 pathways were closely related to inflammation (Fig. 3a). Besides inflammation, biological network analysis showed that the gene set was also enriched for DDR and cellular metabolism (Fig. 3b), which are senescence features in neurodegenerative diseases [9]. Further evaluation of the transcriptome identified 316 significantly differentially expressed metabolism-related genes. Surprisingly, deeper exploration showed that a cluster of molecules consistent with those in neurodegeneration diseases was enriched (Fig. 3c, d), which added to the evidence that IR induces a senescence phenotype in microglia. Next, to verify the reliability of these three distinct features in irradiated microglia, upregulated genes in the transcriptome profile related to the NF-κB pathway (inflammation), apoptosis/cell cycle (DDR), and mitochondrial function (metabolism) were chosen for qRT-PCR confirmation. The result showed that the relative expression of these genes in BV2 cells was dramatically increased at 24 h after IR delivered using linac (Fig. 3e–g). Together, these results indicated that the three identified molecular features (inflammation, DDR, and metabolism) could represent the senescent phenotype of irradiated microglia.

### Differentially expressed LncRNAs in irradiated microglia

**Fig. 4:**
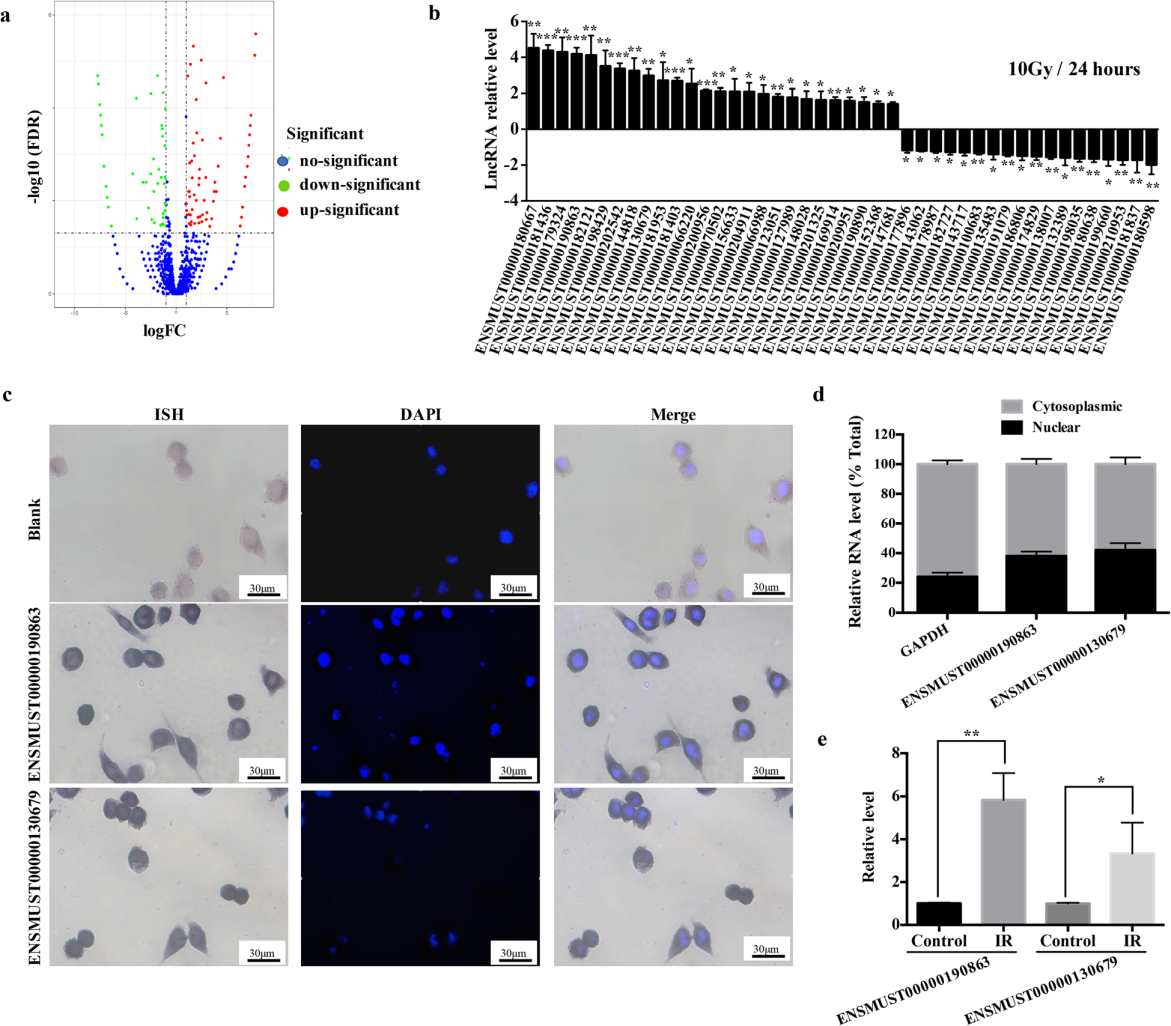
Differentially expressed LncRNAs in irradiated microglia. **a** To identify lncRNAs with altered expression in irradiated microglia, an RNA-seq analysis in BV2 cells was performed. A volcano plot shows the relationship between the magnitude and *q* values of the differences in the control and linac (10 Gy) groups. **b** Expression levels of top 26 upregulated lncRNAs and top 18 downregulated LncRNAs in BV2 cells, as verified using qRT-PCR at 24 h after exposure to linac (10 Gy) (mean ± SEM, *P < 0.05, **P < 0.01, ***P < 0.001). **c** BV2 cells were labeled with ENSMUST00000190863 or ENSMUST00000130679 probes (purplish-blue) using in situ hybridization (ISH), nuclei (blue) were stained using DAPI. Scale bar is 30 μm. **d** qRT-PCR analysis of RNAs purified from nuclear and cytoplasmic compartments in BV2 cells (mean ± SEM). **e** Effect of IR on selected LncRNAs (ENSMUST00000190863 and ENSMUST00000130679) of microglia isolated from the brain tissue of C57Bl6/J mice 24 h after IR, as determined using qRT-PCR (mean ± SEM, *P < 0.05, **P < 0.01)

A number of transcription factors and post-transcription factors have been implicated to drive senescence. As regulators of both transcriptional and post-transcriptional processes, lncRNAs have been demonstrated to have direct regulatory roles in senescence [18]; however, their roles in IR-induced senescence of microglia remain unexplored. Therefore, RNA-seq analysis of lncRNAs in non-irradiated/irradiated BV2 cells was carried out. We produced a volcano plot representing 10919 lncRNAs, of which 84 were upregulated (red plots) and 64 were downregulated (green plots) (*q* < 0.05) (Fig. 4a). Using more stringent criteria, the threshold for differential expression between the control and IR group was set as a log_2_ fold change >2 for lncRNAs. A total of 44 lncRNAs were identified, of which 26 were significantly upregulated and 18 were significantly downregulated. To validate the accuracy of RNA-seq data, qRT-PCR was used to test the expression of these RILs before and after IR. As shown in Fig. 4b, the changes in the expression levels of these lncRNAs were consistent with the RNA-seq profile. Next, two upregulated RILs, ENSMUST00000190863 and ENSMUST00000130679, were selected, both of which were located in the nucleus and cytoplasm of microglia (Fig. 4c, d). In addition, to characterize whether the selected RILs were functional in vivo, primary microglia were isolated from adult mice at 24 h after 20 Gy IR. Both RILs showed significantly increased expression in irradiated microglia (Fig. 4e), suggesting that these two RILs contributed to senescence in vivo. These data indicated that the selected RILs might be functional in the senescent process of irradiated microglia.

### The reciprocal relationship between inflammation and the RILs

**Fig. 5:**
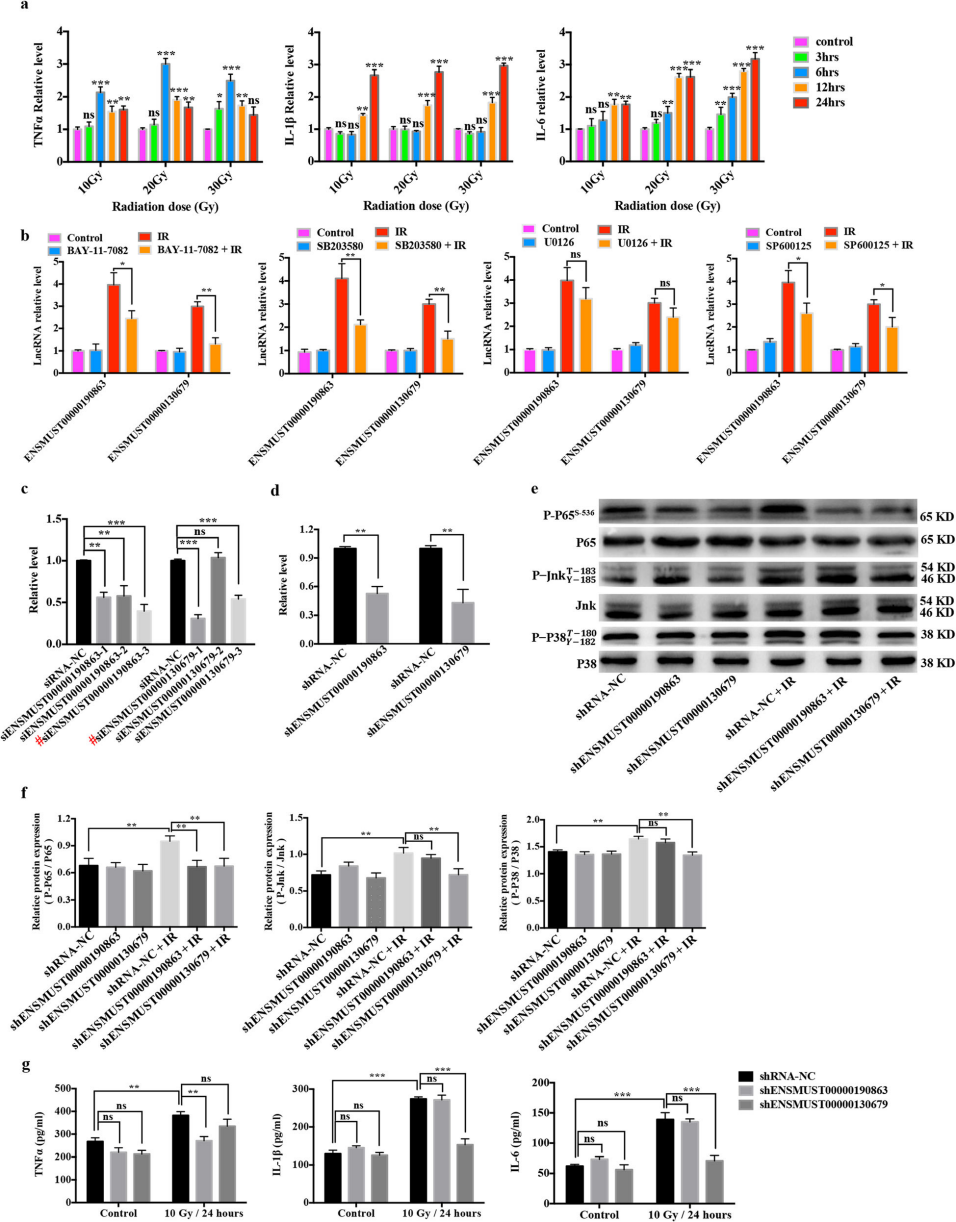
The reciprocal relationship between inflammation and the RILs. **a** Inflammatory cytokine expression in BV2 cells treated with 10 Gy, 20 Gy, and 30 Gy IR using a linac at different time points (mean ± SEM, ns, not significant, *P < 0.05, **P < 0.01, ***P < 0.001). **b** BV2 cells were pretreated with Bay-11-7082 (0.625 μM), SP600125 (2.5 μM), U0126 (5 μM), or SB203580 (10 μM) at 4 h before IR using a linac (10 Gy). Relative expression levels of lncRNAs ENSMUST00000190863 and ENSMUST00000130679 were evaluated at 24 h after IR using qRT-PCR (mean ± SEM, ns, not significant, *P < 0.05, **P < 0.01). **c** qRT-PCR assay of the efficacy of expressing siRNA targeting RILs or NC in BV2 cells (mean ± SEM, ns, not significant, **P < 0.01, ***P < 0.001). Hash (#) represents the most effective sequences (ENSMUST00000190863-3 and ENSMUST00000130679-1) which were chosen for further study. **d** The chosen sequences were used to construct of recombinant virus. qRT-PCR assay of the efficacy of expressing shRNA targeting RILs or NC in BV2 cells (mean ± SEM, **P < 0.01). **e** The phosphorylation levels of P65 (P-P65), Jnk (P-Jnk), and p38 (P-P38) after treatment with shRNAs targeting RILs or NC at 4 h after exposure to 10 Gy using a linac in BV2 cells, as revealed using western blotting. **f** Histogram of the quantification of the immunoreactive protein bands on the western blots (mean ± SEM, ns, not significant, **P < 0.01). **g** The effects of ENSMUST00000190863 or ENSMUST00000130679 knockdown on the expression levels of inflammatory cytokines (TNFα, IL-1β, and IL-6) secreted by BV2 cells at 24 h after 10 Gy IR using a linac, as measured using ELISA (mean ± SEM, ns, not significant, **P < 0.01, ***P < 0.001)

We next investigated whether the RILs can interplay with inflammation, the first identified feature of senescence in irradiated microglia. First, we measured the expression levels of TNFα, interleukin (IL)-1β, and IL-6 after treatment with different doses of IR delivered using linac at different time points. IR remarkably enhanced the mRNA expression levels of *Tnfa*, *Il1b*, and *Il6* in a dose- and time-dependent manner (Fig. 5a). In addition, previous studies proved that the inflammatory state of microglia after IR activation was related to distinct signaling pathways, such as the NF-κB and MAPK pathways [26, 27]. Therefore, selective pharmacological inhibitors of the NF-κB pathway (Bay-11-7082) and the three main branches of MAPK pathway, including SP600125 (c-Jun N-terminal kinase, JNK), U0126 (extracellular signal-regulated kinase, ERK), and SB203580 (p38) were used to detect the possible pathways that act on the expression of the two selected RILs. The result showed that pretreatment with Bay-11-7082 (NF-κB), SP600125 (JNK), and SB203580 (p38) exerted partial inhibitory effects on IR-induced RIL expression (Fig. 5b). These findings suggested that the NF-κB, JNK, and p38 pathways are responsible for the upregulation of the selected RILs in irradiated microglia.

Next, we investigated whether the selected RILs have a bearing on the activation of the NF-κB, JNK, or p38 pathways and further influence the release of pro-inflammatory cytokines. As presented in Fig. 5e, f, the phosphorylation levels of critical signaling proteins P65 (P-P65), JNK (P-JNK), and p38 (P-P38) were significantly upregulated after 10 Gy delivered using a linac in the treatment of BV2 cells. Importantly, silencing the expression of ENSMUST00000190863 attenuated the phosphorylation level of P65, while silencing the expression of ENSMUST00000130679 decreased the phosphorylation level of P65, JNK, and P38 induced by IR (Fig. 5e, f). Furthermore, silencing of the two selected RILs did not change the basal levels of inflammatory cytokines (Fig. 5g); however, knockdown of ENSMUST00000190863 significantly decreased the level of TNFα triggered by IR (Fig. 5g left), while the levels of IL-1β and IL-6 decreased significantly after ENSMUST00000130679 was knocked down in the IR group (Fig. 5g middle and right). These data indicated that the inflammatory state affected the expressions of RILs, while conversely, the RILs could regulate the phosphorylation level of critical signaling proteins in the IR-induced inflammatory process and the downstream release of pro-inflammatory cytokines.

### The reciprocal relationship between the DDR and RILs

**Fig. 6:**
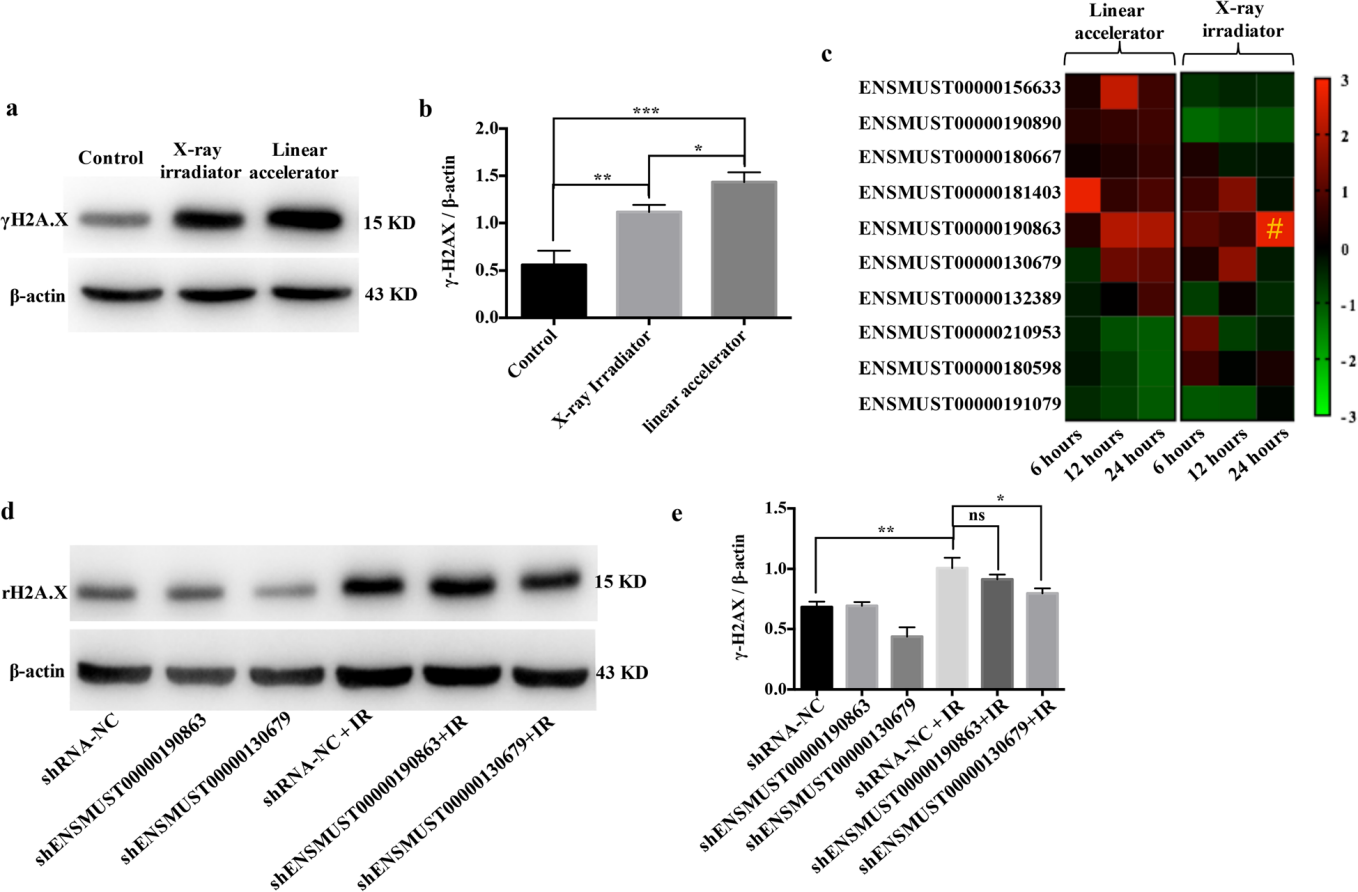
The reciprocal relationship between DNA damage and the RILs. **a** The protein levels of γh2A.X in BV2 cells a 1 h after exposure to 10 Gy using a linac or X-ray irradiation, as revealed using western blotting. **b** Histogram of the quantification of the immunoreactive protein bands on the western blot (mean ± SEM, *P < 0.05, **P < 0.01, ***P < 0.001). **c** BV2 cells were exposed to 10 Gy using a linac or X-ray irradiation, and the relative expression of ten randomly selected RILs from the RNA-seq data (seven upregulated and three downregulated) at different time points were measured using qRT-PCR. The relative expression levels were normalized using the *Z*-score and presented in a heatmap. Hash (#) represents the only RIL in X-ray irradiation group that shared the same expression trend with RNA-seq. **d** The protein levels of γh2A.X in cells treated with shRNA targeting RILs or NC at 1 h after exposure to 10 Gy using a linac in BV2, as revealed using western blotting. **e** Histogram of the quantification of the immunoreactive protein bands on the western blot (mean ± SEM, ns, not significant, *P < 0.05, **P < 0.01)

Different types of radiation with different energy levels vary in their linear energy transfer (LET) properties. With increasing LET, the severity of the DDR increases [28]. To investigate whether RILs can interplay with the DDR, the second identified feature of senescence in radiated microglia, we used different radiation energies at the same dose to trigger different DDRs. As expected, compared with the 225 KV X-ray irradiator, BV2 cells accumulated higher levels of the DDR marker protein γH2A.X when using a 6 MV linac (Fig. 6a, b), indicating that a more severe DDR is induced at higher radiation energy. To investigate the impact of different DDR levels on the expression of RILs, seven upregulated and three downregulated RILs, including the selected ENSMUST00000190863 and ENSMUST00000130679, were chosen and validated using qRT-PCR. As shown in the heatmap (Fig. 6c), all these candidates showed the same expression profile after higher radiation energy exposure using linac at 24 h between the RNA-seq and qRT-PCR data (Fig. 6c left); however, only ENSMUST00000190863 had the same trend after lower radiation energy exposure using the X-ray irradiator (Fig. 6c right). One possible explanation is that different severities of DDR would result in variations in the expression profiles of the RILs.

To identify the possible regulatory roles of the two selected RILs in the severity of DDR, western blotting was performed. The upregulation of γH2A.X triggered by 10 Gy using linac was attenuated when ENSMUST00000130679 was silenced, but not when ENSMUST00000190863 was silenced (Fig. 6d, e). These results demonstrated that the severity of DDR affected the expression of RILs. In turn, certain RILs have the ability to influence the severity of DDR.

### The reciprocal relationship between lipid metabolism and the RILs

**Fig. 7:**
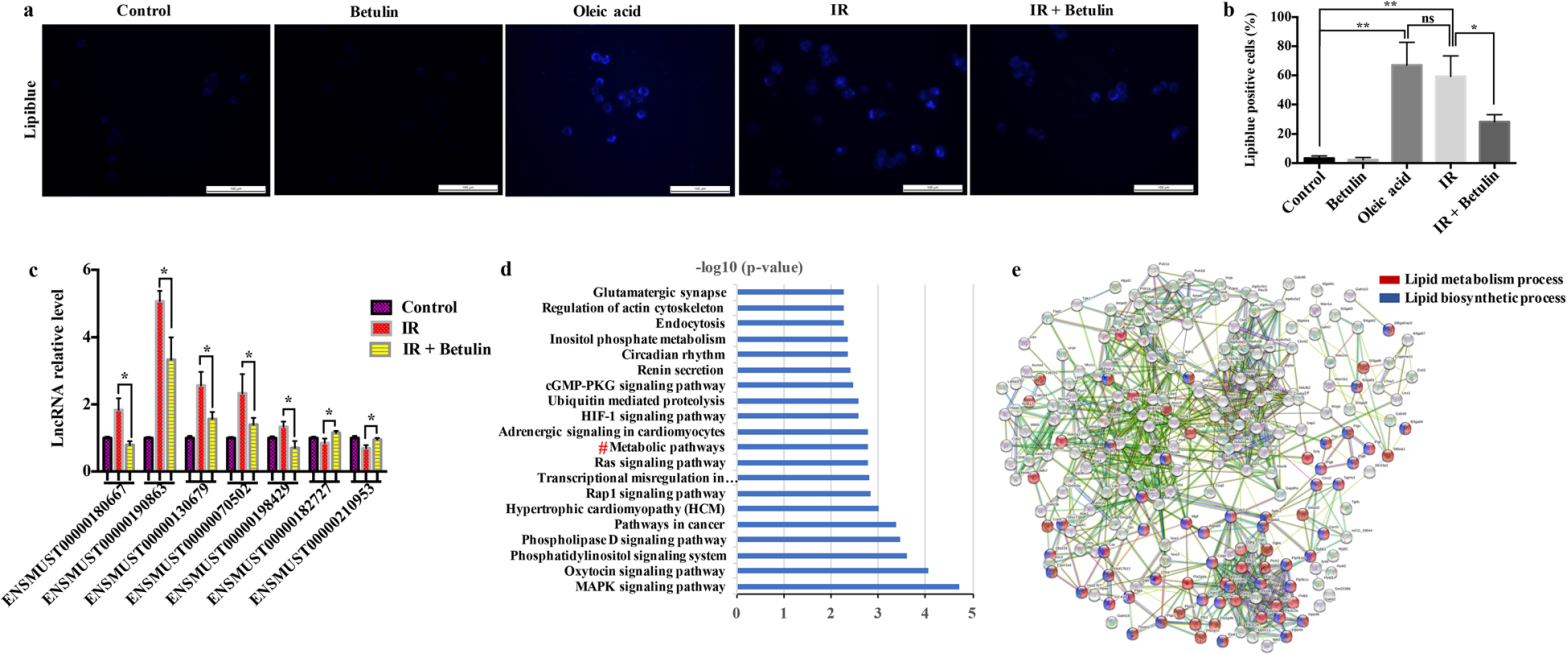
The reciprocal relationship between lipid metabolism and the RILs. **a** BV2 cells were exposed to 10 Gy using a linac at 5 h after treatment with/without 2 μM betulin. Representative micrographs of lipiblue staining in BV2 cells at 24 h after IR exposure. 100 μM Oleic acid (OA) treatment was used as the positive control. Scale bar = 100 μm. **b** Quantification of lipiblue positive cells (mean ± SEM, ns, not significant, *P < 0.05, **P < 0.01). **c** The relative expression levels of randomly selected RILs in BV2 cells at 24 h after exposure to 10 Gy using a linac with/without betulin (2 μM) (mean ± SEM, *P < 0.05). **d** KEGG was used to cluster 3589 neighboring protein genes of RILs (neighboring genes within 20 kilobase of the 84 upregulated and 64 downregulated RILs (FDR *q* value <0.05) in RNA-seq data). Hashes (#) represent 226 metabolism-related genes. **e** GO analysis identified 79 lipid metabolism-related genes (red), and 45 lipid biosynthetic metabolism-related genes (blue) from the RILs’ neighboring protein genes

The third identified molecular feature of senescence in irradiated microglia was cellular metabolism. Recent studies showed that lipid droplets (LDs) accumulate in microglia during the aging process [29, 30]. To detect whether LD accumulation also occurs in IR-induced senescent microglia, we exposed BV2 cells to a 10 Gy single-dose delivered using linac and examined LDs using lipiblue staining. The result showed a significantly increased number of LDs in irradiated BV2 cells compared with that in the control cells, similar to those stimulated by OA as a positive control. Surprisingly, after pretreatment with betulin, a sterol regulatory element-binding protein (SREBP) inhibitor, the number of LDs in irradiated BV2 cells decreased significantly (Fig. 7a, b). This suggested that the accumulation of LDs in microglia is caused by the activation of lipogenesis after IR. Next, qRT-PCR was used to test whether lipogenesis affected the expression of RILs. As shown in Fig. 7c, the lipogenesis inhibitor betulin significantly attenuated the expression changes of RILs triggered by IR (including ENSMUST00000190863 and ENSMUST00000130679).

LncRNAs frequently exert their biological functions by regulating the expression of nearby genes [31]. Therefore, we examined 3589 neighboring genes using KEGG analysis and identified 226 genes related to cellular metabolism (Fig. 7d). Among them, 79 were lipid metabolism-related, and more than half of these (45/79) were lipogenesis- related (Fig. 7e). Collectively, our data suggested that lipogenesis is activated by IR, which triggers changes in the expression levels of RILs. In turn, RILs might function in lipogenesis via the effects of their neighboring genes.

### Blockade of the RILs alleviated neuronal damage after radiation

**Fig. 8:**
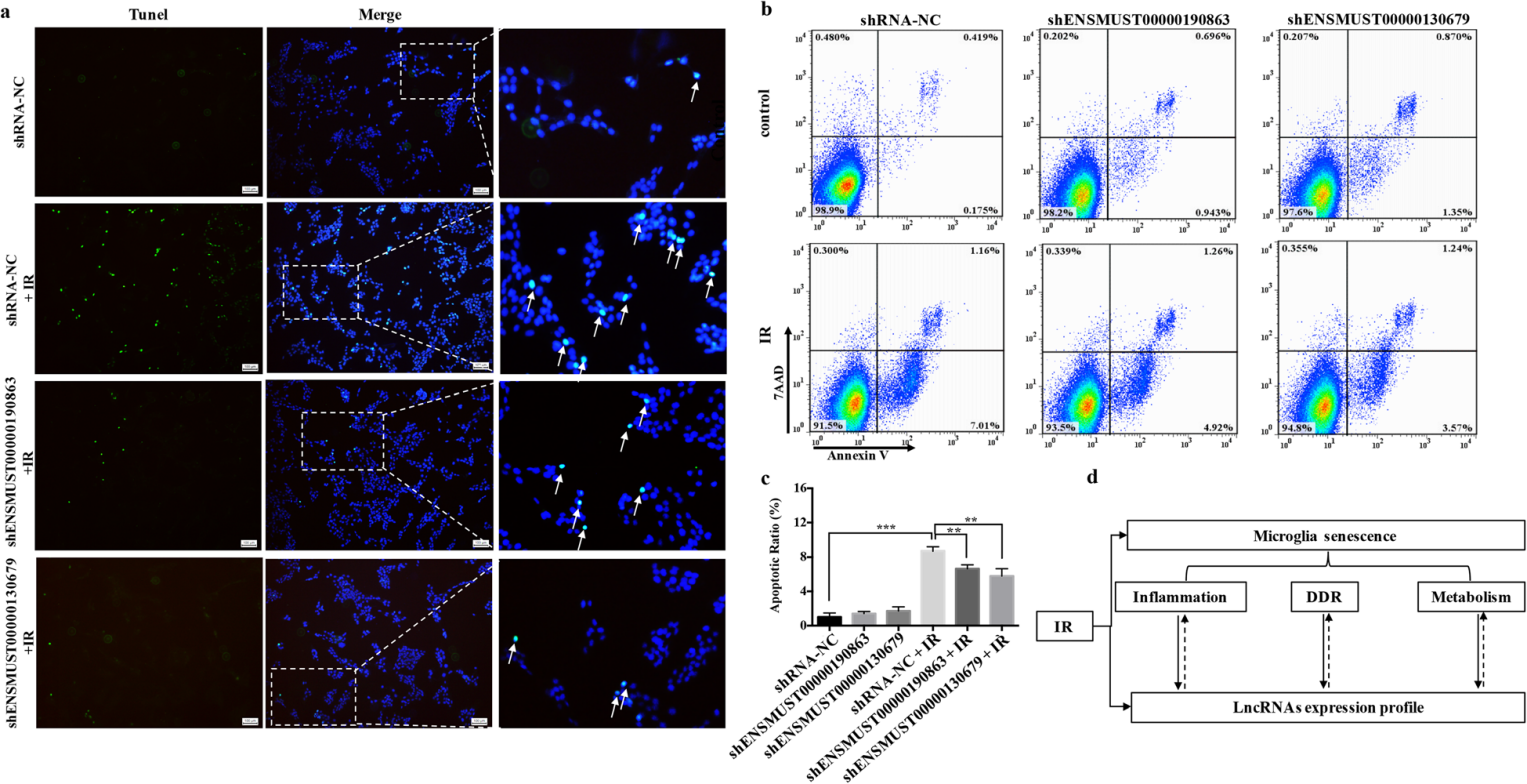
Blockade of RILs alleviated neuronal damage after radiation. **a** Neuron HT22 cells were co-cultured with irradiated BV2 cells with/without ENSMUST00000190863 or ENSMUST00000130679 knockdown for 24 h. Apoptotic HT22 cells were identified using TUNEL staining. Representative micrographs are shown. Scale bar = 100 μm. **b** Apoptotic HT22 cells were quantified using flow cytometry with Annexin V-APC/7-AAD staining. **c** The ratio of apoptotic cells is shown (mean ± SEM, **P < 0.01, ***P < 0.001). **d** Schematic diagram of the reciprocal relationship between the senescence phenotype and RILs in radiation-treated microglia

To determine whether inhibiting the expression of the two selected RILs associated with senescence in IR-induced microglia could affect neuronal damage, irradiated BV2 cells were co-cultured with the mouse neuron cell line HT22 in a Transwell system. Apoptotic neuronal cells were detected using TUNEL staining and quantified using flow cytometry. The apoptosis ratio of HT22 cells increased remarkably after co-culture with irradiated BV2 cells (Fig. 8a–c). Surprisingly, this ratio decreased dramatically when ENSMUST00000190863 or ENSMUST00000130679 were inhibited (Fig. 8b, c). In summary, these findings indicated that neuronal damage caused by irradiated microglia could be alleviated by downregulating the expression of certain RILs.

## Discussion

In this study, we showed that microglia acquired a senescence phenotype after IR. Analysis of the transcriptome of irradiated microglia identified three main molecular features of senescence, including inflammation, the DDR, and metabolism. Our findings also showed that RILs are closely related to the molecular features of the senescence phenotype in irradiated microglia (Fig. 8d).

The molecular characteristics of primary microglia at 24 h and 1-month post-irradiation in adult mice share similarities with primary microglia in aging mice [16]. In this study, we found that the two key markers of senescence, SA-β-Gal and p16^INK4A^, were both elevated dramatically in irradiated microglia. We also observed the persistence of β-galactosidase activity and the p16^INK4A^ signal in microglia at 1-month post-irradiation in vivo. Chronic senescence is believed to be a driver of age-related tissue dysfunction. The gradual accumulation of senescent cells alters the physiological function of tissues and organs, ultimately leading to age-related diseases [23, 32]. Microglial senescence has profound consequences for neuronal activity and cognitive function in the normal aging brain [10], and irradiated microglia play a pivotal role in the underlying pathology of RIBI [7]; therefore, we speculated that the senescence induced in irradiated microglia might also explain the observed neurotoxic effects. In addition, the two main signaling pathways, p53-p21-RB and p16^INK4A^-RB, are coordinated to initiate and maintain senescence [23]. Interestingly, we detected a significant increase in p16^INK4A^ in irradiated microglia, whereas the expression levels of p53 and p21 remained unchanged. In addition, the p53 signal was not detected after DNA damage in irradiated astrocytes [33]. Furthermore, a significant role of p16^INK4A^ was demonstrated in p53-independent senescence of primary human fibroblasts [34]. Accordingly, we deduced that cellular senescence in irradiated microglia occurs in a p53-independent manner and that p16^INK4A^-RB signaling might be the major functional pathway.

Increasing evidence has demonstrated sex-specific differences in normal aging microglia [35]. Consistently, sex-specific differences also exist in irradiated microglia, including microglia-mediated-inflammation, synaptic modification, and cognitive deficit [36, 37]. For example, the sex-specific difference in irradiated microglia-mediated dendritic spine pruning was derived from a sex-specific difference in the expression of complement receptor 3 (CR3) [37]. We speculated that the phenotype of irradiation-induced microglial senescence found in the current study might also vary with sex, which is worthy of further exploration.

In senescent cells, characteristic molecular features have been observed [9]. In the present study, we confirmed the three main molecular features of senescence in the transcriptome of irradiated microglia as inflammation, the DDR, and metabolism. Sustained abnormal activation of microglia is thought to contribute to a chronic inflammatory state in the brain [38], which shares similarity with the role of microglia in neurodegenerative diseases [6]. In the present study, we demonstrated that the expression of genes related to NF-κB, the central regulator of inflammation, were upregulated significantly after IR treatment. Secondly, a sustained DDR could be detected 1 month after IR in primary microglia [16]. Typically, DNA damage is resolved within hours; however, severe or irreparable DNA damage causing persistent DDR signaling is essential to establish and maintain a senescent phenotype [39]. Therefore, we measured the expression level of target genes of apoptosis and the cell cycle, the downstream mediators of DDR signaling, and demonstrated that the expression levels of these genes remained upregulated in microglia at 24 h after IR exposure. Thirdly, aged brains suffer from metabolic reprogramming [40]. Given the critical role of mitochondria in regulating metabolism, we examined the expression of genes related to mitochondrial function in the microglia and observed their increased expression after IR. Taken together, these findings provide evidence that IR induces changes to inflammation, the DDR, and metabolism that are senescence-specific.

LncRNAs have emerged as major regulators of genomic functions during aging [41]. In addition, senescence-associated lncRNAs (SAL-RNAs) play direct regulatory roles in senescence [18]. Moreover, lncRNAs are abundantly expressed in cells of the CNS [42]. However, the function of RILs in senescent microglia remains unexplored. Our result revealed that the expression levels of the two selected RILs were increased significantly in irradiated microglia, suggesting their potential roles in regulating gene expression during senescence in irradiated microglia.

As an important feature of microglia senescence, inflammation is crucial in IR-induced RIBI [38] and aging-related diseases [6]. Abnormally activated microglia constantly produce neurotoxic cytokines, such as IL-1β, TNFα, and IL-6, and increase the level of reactive oxygen species (ROS), which consequently induce oxidative stress, ultimately leading to DNA damage [25]. In the present study, inflammatory cytokines were released persistently by microglia after IR and increased in an IR dose-dependent manner. In addition, in peripheral macrophages, lncRNAs are induced or suppressed after the activation of intracellular signaling pathways, and these alterations to lncRNA levels promote, restrain, or suppress the response of immunity-related genes [43–45]. Accordingly, we suspected that the role of RILs in irradiated microglia might resemble the regulation process in macrophages. Therefore, the classical inflammatory pathways, NF-κB and MAPK, were evaluated [26, 27]. We found that activation of NF-κB, JNK, or p38 pathways regulated the expression of selected RILs, which in turn influenced inflammation by regulating the phosphorylation levels of critical signaling proteins.

The DDR is the second important molecular feature of radiation-induced senescent microglia. Our results showed that the severity of the DDR was affected by radiation energy. The amount of energy that an ionizing particle transfers to the material traversed per unit distance is defined as the LET. High LET tends to mediate direct radiation interaction damage, while low LET mainly mediates indirect oxidative damage via water radiolysis [28, 46]. Unrepaired or inaccurately repaired DNA damage can induce cells to activate the death mechanism or acquire other states, such as senescence, which lead to permanent functional changes [47]. γH2AX is the most sensitive marker to examine the DDR [48]. Distinct expression profiles of RILs were associated with different levels of γH2A.X upregulation induced using different IR energies. In addition to key protein-coding genes, a subset of non-coding RNAs is also required for the cellular DDR. To coordinate the non-coding RNA-mediated DDR, DNA damage can regulate the expression levels of non-coding RNAs. In turn, lncRNAs participate in regulating certain steps of the DDR process, including recognition of DNA damage, signal relays, and the initiation of the repair [49]. Similarly, our result showed that the silencing of ENSMUST00000130679 resulted in decreased γH2A.X levels. This suggested that ENSMUST00000130679 participates in the DDR process. These findings will contribute to a better understanding of the reciprocal relationship between the radiation-induced DDR and RILs.

We detected the metabolism-related genes aggregation using RNA-seq and the accumulation of LDs in the early stage of microglia irradiation. A recent study confirmed that the accumulation of LDs in microglia is a marker of a dysfunctional state of the aging brain [30]. Furthermore, elevated ROS levels, followed by mitochondrial dysfunction, cause lipid peroxidation, which triggers the accumulation of LDs in glia. Reducing LD accumulation and lipid peroxidation in glia significantly delayed the onset of neurodegeneration [50]. Interestingly, we found that the accumulation of LDs decreased when lipogenesis was blocked in irradiated microglia. This suggested that enhancing lipogenesis, rather than inhibiting lipolysis, is the main cause of LD accumulation in irradiated microglia. Therefore, we further evaluated the roles of RILs in lipogenesis of irradiated microglia. The upregulation of several RILs after IR was significantly attenuated when lipogenesis was blocked, indicating that certain RILs are involved in lipogenesis. Given that most lncRNAs have varying degrees of overlap with nearby protein-coding genes, compared with the lncRNA itself, the transcriptional activity within the locus containing the lncRNA is more likely to be the source of functional regulation [31]. Therefore, it is reasonable to hypothesize that RILs regulate lipogenesis via nearby genes. Using GO analysis, we showed that 79 out of 226 metabolism-related genes were involved in lipid metabolism. Surprisingly, over half of them were related to lipogenesis. Although we could not conclude directly which lipogenesis-related genes are affected by the RILs, we could speculate that the RILs regulate LD accumulation at least partly via their effects on nearby lipogenesis-related genes.

As the first-line defense against injury, intact microglia undergo “beneficial activation” in the context of brain injury, in which they migrate to the injury, phagocytose the debris, secrete various cytokines and chemoattractants, and eliminate inflammation, eventually resulting in tissue recovery. However, once the activation passes the threshold of beneficial function and becomes deleterious, microglia swiftly acquire a neurotoxic phenotype, causing impairment to phagocytosis and synaptic plasticity, the generation of ROS, and inflammatory disorder, which together contribute to neuronal damage or death [6]. To test the impact of irradiated-induced senescent microglia on neurons, we built a co-culture system of BV2 and HT22 cells. The two selected RILs had a significant impact on the apoptosis ratio of HT22 cells, which suggested that RILs might be novel prognostic biomarkers and potential targets for protection against encephalopathy.

## Conclusions

In summary, our study provides new insights into the pathophysiology of irradiated microglia. Inflammation, the DDR, and metabolic changes are the three principal molecular features of senescence in irradiated microglia, and distinct RILs are closely associated with these molecular features. Increased understanding of the functions of RILs will reveal their potential therapeutic value in RIBI.

## Supplementary Information

**Additional file 1: Supplementary Table 1.** qRT-PCR primers used.

## Data Availability

All data in this manuscript are available on reasonable request.

## Abbreviations

RIBI: Radiation-induced brain injury
lncRNAs: Long noncoding RNAs
DDR: DNA damage and response
RILs: Radiation-induced differentially expressed lncRNAs
IR: Ionizing radiation
QoL: Quality of life
DAMPs: Damage-associated molecular patterns
MMP: Matrix metalloproteases
LDs: Lipid droplets
FC: Fold-change
OA: Oleic acid
D-PBS: Dulbecco’s phosphate-buffered saline
LPS: Lipopolysaccharide
ISH: In situ hybridization
SSC: Saline sodium citrate
SA-β-Gal: Senescence-associated β-galactosidase
Iba-1: Ionized calcium-binding adapter molecule 1
Linac: Linear accelerator
Dpi: Days post-irradiation
RNA-seq: RNA-sequencing
FDR: False discovery rate
GO: Gene ontology
KEGG: Kyoto Encyclopedia of Genes and Genomes
CDKi: Cyclin-dependent kinase inhibitor
Jnk: C-jun N-terminal kinase
Erk: Extracellular signal-regulated kinase
IL-1β: Interleukin-1β
TNFα: Tumor necrosis factor α
LET: Linear energy transfer
SREBP: Sterol regulatory element-binding protein
ROS: Reactive oxygen species
ELISA: Enzyme-linked immunosorbent assay
qRT-PCR: Quantitative real-time reverse transcription-polymerase chain reaction
