# Supplementary material for: Regulatory coupling between long noncoding RNAs and senescence in irradiated microglia
Source: J Neuroinflammation. 2020 Oct 28;17:321. doi: 10.1186/s12974-020-02001-1 (PMC7592596; doi:10.1186/s12974-020-02001-1)
Supplement: Supplementary file 1 — Additional file 1: Supplementary Table 1. qRT-PCR primers used. [file 12974_2020_2001_MOESM1_ESM.docx]

**Supplementary Table 1. qRT-PCR primers used**

| **Gene** | **Forward primer (5’-3’)** | **Reverse primer (3’-5’)** |
| --- | --- | --- |
| **MMP3** | CCTGATGTTGGTGGCTTCA | TCCTGTAGGTGATGTGGGATTTC |
| **P16^INK4a^** | AATCTCCGCGAGGAAAGC | GTCTGCAGCGGACTCCAT |
| **P53** | CAGGCACAAACACGAACC | ATGAACCGCCGACCTATC |
| **P21** | TTGCCAGCAGAATAAAAGGTG | TTTGCTCCTGTGCGGAAC |
| **TNFα** | CCTCCACTTGGTGGTTTGT | GGCGGTGCCTATGTCTCA |
| **IL-1β** | CTTCTCCACAGCCACAAT | AGCATCCAGCTTCAAATC |
| **IL-6** | GACTCTGGCTTTGTCTTT | GGCAATTCTGATTGTATG |
| **Cox2** | TGCCCACAACTGTCTTCCAT | CTGATCGGCGTGACTACCC |
| **Arg1** | AGTCAGTCCCTGGCTTAT | AAGACAGCAGAGGAGGTG |
| **IL-10** | GGATCATTTCCGATAAGG | TTTCAAACAAAGGACCAG |
| **Itgam** | ATGGACGCTGATGGCAATACC | TCCCCATTCACGTCTCCCA |
| **Cx3cr1** | GAGTATGACGATTCTGCTGAGG | CAGACCGAACGTGAAGACGAG |
| **CD68** | TGTCTGATCTTGCTAGGACCG | GAGAGTAACGGCCTTTTTGTGA |
| **Ltb** | TGGCAGGAGCTACTTCCCT | TCCAGTCTTTTCTGAGCCTGT |
| **CXCL2** | CCAACCACCAGGCTACAGG | GCGTCACACTCAAGCTCTG |
| **Traf1** | AGGGTGGTGGAATTACAGCAA | GCAGTGTAGAAAGCTGGAGAG |
| **ptgs2** | TGCACTATGGTTACAAAAGCTGG | TCAGGAAGCTCCTTATTTCCCTT |
| **Lck** | ATTGACGTGTGTGAAAACTGCC | ATCCCTCATAGGTGACCAGTG |
| **LBP** | GTCGTGGGCAGTACGAGTTTC | CTCGGACACCGATGGAAGA |
| **CD40** | TGTCATCTGTGAAAAGGTGGTC | ACTGGAGCAGCGGTGTTATG |
| **Ddx58** | GAAGAGCCAGAGTGTCAGAATC | AGCTCCAGTTGGTAATTTCTTGG |
| **Lat** | TAGTCACTTCCTTCCCACCC | ACTGTCAGGCAGCACCACT |
| **Thbs1** | GATCAGCAAATCTCTCCAAAACC | CCACCTAATTCCACTTGAAACTACA |
| **Serpine1** | CCTTGCTTGCCTCATCCTGG | TTTTCCCCGCTGTGGTCATC |
| **Serpinb5** | TCAAAGACAAACTGGAAGAAACG | AGGCAGCATTAACCACAAGGA |
| **Cdk6** | GGACATCATTGGACTCCCAGGA | GGATTAAACGTCAGGCATTTCAGAA |
| **Apaf1** | GCGACCAAGTTTTCATCTACCT | GATTTCTCCATTGTCATCTCCAG |
| **Mdm2** | AGGCAGAAGAAGGCTTGGATGT | TGGAAGTCGATGGTTGGGAATA |
| **Rfwd2** | ATCAAAGCAGATGGGACAGA | CTCGGCAGTAGCAGTAGGAA |
| **Shisa5** | AGCCAGAGTCCAGCAGATTT | CAGGTGAAGCAGATGATGATAGT |
| **CCnb1** | CGTGACAAAGGCATAACTCCAATAG | GCCTAAACTCAGAAGCAACAACATT |
| **CCNE2** | TGGTGCCTTTTGTTAGTGTTG | TCAGTGTTTTCCTGGTGGTTT |
| **NDUFA10** | CCGCCTTCTTCAGTATGCAGA | TGCTTTCGGATATAGCCCTGG |
| **NOS1** | ACGGCAAACTGCACAAAGC | CGTTCTCTGAATACGGGTTGTTG |
| **PLCB4** | GGACAAGTGCTAGAATGTTCCC | GAAGCCGATATTCACCAGATCC |
| **ATP5A1** | TCTCCATGCCTCTAACACTCG | CCAGGTCAACAGACGTGTCAG |
| **COX5B** | GGAAGACCCTAATCTAGTCCCG | GTTGGGGCATCGCTGACTC |
| **CYCS** | CCAAATCTCCACGGTCTGTTC | ATCAGGGTATCCTCTCCCCAG |
| **ATP5H** | ATGGCTGGGCGTAAACTTG | CTGGCGTGGAAGGTCTCATT |
| **ATP5G3** | CTGGTATTGGAACAGTCTTTGGC | GATCAAGAACGCAACCATCAAAC |
| **NDUFS1** | TAGCAAATCACCCATTGGATTGT | CACCGGGTACACTGGATGC |
| **NDUFB5** | CAAGAGACTGTTTGTCGTCAAGC | TGTTCACCAGTGTTATGCCAAT |
| **ENSMUST00000066220** | TCGGACGGCACAGAGGACTTT | CCAGAACGGAGGGGTAGAGCA |
| **ENSMUST00000127989** | TATGGTGGAATGAGGTGGTTG | TGGAGATACGGAAGGTTGTGA |
| **ENSMUST00000152368** | ATGCTTGATTCTCCTCTTCCTG | GGCTTCTGATTTCACCGTACA |
| **ENSMUST00000156633** | TGGTGGATAACTCGGTGGTAG | CAAGACCCATCCTGACCTGTG |
| **ENSMUST00000190890** | AGGGCATCACTGAAGGAAACG | GGACCTGACGACCCAACAAAG |
| **ENSMUST00000066988** | GCATTTAGGAGGCTGTCATCT | GAAGTCAACGGCTCTGTAGGT |
| **ENSMUST00000169914** | TTACCAGGGAGGTGGGTGTTT | TCCGAGTCTCCGCAGTTCATC |
| **ENSMUST00000182121** | AACCATTTTCCCTCCTTGACC | TGACCACCACATCCACCACTA |
| **ENSMUST00000180667** | TTTGTCCCAGAGCAATGTATG | ACAGTGCGGTGTAGTATGAGG |
| **ENSMUST00000202542** | TGTTGATCTGAAAGGAAGTGG | TTGGACTGATGACTGACGACT |
| **ENSMUST00000204911** | TAGCTGGGTTTGGTAGAGTGC | TGGGCAGAGTCTCATGTATCC |
| **ENSMUST00000179324** | AGGACTGAAACTCAAAACCCA | GAAACCACCTTTAGCCATCAT |
| **ENSMUST00000181403** | GTTATGTGCAAGGGTCAAAGT | TCAAGAGTTCAAGAGGGGTTC |
| **ENSMUST00000148028** | CTGTTGGGCTTCTTCATTGCT | CATTCAGGGACCGAGGATAGG |
| **ENSMUST00000070502** | TCCATTGCCTCATCCCATCTT | TTTTCCCAGTTCCCTTACGCC |
| **ENSMUST00000198429** | CCTTATCTCAGCGTCAAAACC | TCCTGGCAACAGTGAAACATT |
| **ENSMUST00000190863** | TTTTCCTGTTTGGTTCTTTGG | TATCTACTCGCCCCTCTGACT |
| **ENSMUST00000201325** | CACCACAATGGCTGTAATCTC | CACAATGCTGGAATGGAACTA |
| **ENSMUST00000181436** | CCAGCGTCCCCAACTTTCTAT | CCTCTGTTCACTTTGGCTCCG |
| **ENSMUST00000147681** | GAATGATAAAAGCAAAGTGGAAA | TGCATAGTGGTACATGAGGGAT |
| **ENSMUST00000144818** | GTCACCACATCTCACCCATCC | CATCCCCATCACAGAAAGCAC |
| **ENSMUST00000123051** | CGAGCTGGTATCGGCTTCTGG | CAGTTGCCCCTGTGGATGTGG |
| **ENSMUST00000130679** | CTGCCAGTAACAGGAAACCGA | ACGCATAGTAGCAAACCCAAAA |
| **ENSMUST00000181953** | TGTCACTGGGGAAGAACTTTG | TTGATTGAGGCTTGCTTATGG |
| **ENSMUST00000209951** | CAGTAGGTCTCCAGCATCACGTCTT | AACACCCCACCACTCGACCTC |
| **ENSMUST00000200956** | AAATGGAAGCCAGAGGGAAGC | CCCACTCACCAAAGGTCAACG |
| **ENSMUST00000199660** | AGCTGTAGCCCAACTCCTCCC | TGCCGACTGCCATTGTAAGAC |
| **ENSMUST00000182727** | CTTGGAACCATTTACTTTGACT | CTCCTTAACCTTTTGGCTTTT |
| **ENSMUST00000143062** | GTCCCCGAGCCTTAGTGGTTT | TGAGCATGGAAATGGCAGAGT |
| **ENSMUST00000135483** | TCTTCGTTCTGCCTCTTTGTC | CCAGGTAGCTCAAGCAATGTC |
| **ENSMUST00000138007** | TTCGGATAGATGGGTTAGACT | TACGATGACTCGGACATACTC |
| **ENSMUST00000132389** | TCGGACGGCACAGAGGACTTT | CCAGAACGGAGGGGTAGAGCA |
| **ENSMUST00000198035** | TGGAAAGGGATACAAGACATA | TTCAGACAAAAGCAGAAGAGG |
| **ENSMUST00000100683** | GAAAGCATCAGGGAAGAGTGT | AAAGAAAACCAGAAGCCAAAA |
| **ENSMUST00000143717** | TTGCCCAAATATCACAAGAGC | CCCTAAAGGAAGCCTGAAAGA |
| **ENSMUST00000181837** | TCCTTGTTCTACCCTGTCTGC | TCCCTTCTTCCCTCTTTCTGG |
| **ENSMUST00000210953** | GTGGGTCATAGCCTGTAGATT | GGAGGTGAGTAGCTCGATTTC |
| **ENSMUST00000180598** | GCTGATGAATCGTATTTGGTG | TTGATGGGAGTGACAGAGGAA |
| **ENSMUST00000174829** | TGTCCTACACCTTACGCAATC | TTCTAAACAAGCCTCCATCCA |
| **ENSMUST00000186806** | CACAGGCTCACAGTCCATTCT | TTTCCGTGGCTAGTAAAGTCC |
| **ENSMUST00000180638** | CCCAATGGAGATTAGGGAGGA | TGACCGACAAGGGATGGACTG |
| **ENSMUST00000178987** | CAATAGGTGTCTGGAGGGTGG | TGTTCTCAGGCGTCGTAGGTA |
| **ENSMUST00000191079** | GCTGGCGTTGGCGGGTAAAGA | CGGAGTGGCATCGCACCTTGT |
| **ENSMUST00000177896** | GGAGTTGGAGATGGAGGGTTG | GGGGAGTGATGCTGGTAGAGG |
| **GAPDH** | AAGAAGGTGGTGAAGCAGG | GAAGGTGGAAGAGTGGGAGT |
